# Exploring the relevance of male involvement in the prevention of mother to child transmission of HIV services in Blantyre, Malawi

**DOI:** 10.1186/s12914-014-0030-y

**Published:** 2014-10-30

**Authors:** Alinane Linda Nyondo, Angela Faith Chimwaza, Adamson Sinjani Muula

**Affiliations:** 1 School of Public Health and Family Medicine, College of Medicine, University of Malawi, Blantyre, Malawi; 2 Kamuzu College of Nursing, University of Malawi, Blantyre, Malawi

**Keywords:** PMTCT, Male involvement, Relevance

## Abstract

**Background:**

Male involvement (MI) in Prevention of mother to child transmission (PMTCT) of Human Immunodeficiency Virus (HIV) services remains low despite the progress registered in the implementation of the PMTCT program. Male involvement in PMTCT is a fairly new concept in Malawi that has not been fully implemented within PMTCT service provision despite its inclusion in the PMTCT guidelines. One of the reasons for the limited MI is the lack of knowledge on both its relevance and the role of men in the program. Currently, men have been encouraged to participate in PMTCT services without prior research on their understanding of the relevance and their role in PMTCT. This information is vital to the development of programs that will require MI in PMTCT. The objective of this study was to explore the views of men, pregnant women and health care providers on the importance and roles of MI in PMTCT services in Blantyre Malawi.

**Methods:**

An exploratory descriptive qualitative study was conducted from December 2012 to January 2013 at South Lunzu Health Centre (SLHC) and its catchment area in Blantyre, Malawi. We conducted 6 key informant interviews (KIIs) with health care workers and 4 focus group discussions (FGDs) with 18 men and 17 pregnant women. Interviews and discussions were digitally recorded and simultaneously transcribed and translated into English. Data were analyzed using framework analysis approach.

**Results:**

The major themes that emerged on the relevance of MI in PMTCT were a) uptake of interventions along the PMTCT cascade b) support mechanism and c) education strategy. Lack of MI in PMTCT was reported to result into non-disclosure of HIV test results and non-compliance with PMTCT interventions.

**Conclusions:**

Male involvement is paramount for the uptake of interventions at the different cascades of PMTCT. The absence of male involvement may compromise compliance with PMTCT interventions.

## Background

Sub ¿ Saharan Africa (SSA) is hugely affected with Human immunodeficiency Virus (HIV) with 92% of HIV infected pregnant women living in the region [1]. The rollout and uptake of Prevention of Mother to Child Transmission (PMTCT) of HIV interventions has progressed both in the antiretroviral (ARV) regimen provided and the uptake of the intervention by women. In 2011, it was estimated that PMTCT service coverage in SSA was at 59% [1]. Prevention of Mother to Child Transmission of HIV services has the following cascades that a pregnant woman has to navigate to achieve effective reduction in MTCT of HIV: antenatally the cascades are as follows; a) attendance to antenatal care, b) offered and ability to take an HIV test, c) undergo staging to determine eligibility for ART (does not currently apply in Malawi with Option B?+?as policy for all pregnant women), d) initiate ART or PMTCT prophylaxis and e) continue with follow up and adherence to ART antenatally. Postnatally the cascades of intervention are a) give birth with a skilled attendant, b) access postnatal care, c) takes an HIV test if not done antenatally, d) determines the infant¿s HIV status, d) initiate ARVs and e) follow up [2].

Prevention of Mother to Child Transmission of HIV services in Malawi are integrated with Antriretroviral Therapy (ART) services and are offered within the Maternal and Child Health (MCH) section [3]. Malawi implemented Option B?+?as a regimen for PMTCT since 2011. This regimen entails triple ARV to pregnant HIV-infected women, irrespective of her CD4 count or clinical stage. The specific antiretroviral (ARV) regimen provided is TDF/3TC/EFV (Regimen 5A) continued for life from the point of diagnosis. All babies born to HIV infected mothers receive NVP once a day, started as soon as possible after birth and continued for 6 weeks [4]. Malawi uses an Opt Out approach (Provider initiated routine antenatal testing with group education in HIV and PMTCT) in HIV testing for all women presenting at the ANC clinics in which the patient must actively refuse the test. Men have criticised the maternal health services, where PMTCT is offered in Malawi for lacking an agenda for them [5]. Although the uptake of PMTCT services in Malawi has steadily progressed as evidenced by the reduction in the rate of mother to child transmission for Malawi from 25% to 16% in 2012 [6]; the progress has been gradual as antiretroviral coverage for HIV infected pregnant women remains below the desired target of 80% [3]. The initial analysis of retention in care under Option B?+?regimen in Malawi, revealed that 17% of the HIV infected women were lost to follow-up 6 months post ARV initiation and more women dropped out of the group that started ARVs to prevent MTCT compared to those that initiated ARVs secondary to a low CD4 count or for their own health [7].

The levels of MI in PMTCT services in Malawi are similar to those in other countries in SSA with rates as low as 3.2% in Malawi [8], 12.5% in Tanzania [9] and 16% in Kenya [10]. One of the reasons for the low rates of MI is the lack of knowledge about the relevance of MI in PMTCT services and the role of men in the service [11],[12]. Male involvement in Antenatal care (ANC) and PMTCT of HIV services resulted in increased uptake of antenatal care services in Nepal and United States of America [12]¿[14], increased uptake and adherence to PMTCT interventions in Tanzania and Kenya [9],[15] uptake of HIV testing by women in Tanzania and Uganda [16],[17] an opportunity of male HIV testing in Burkina Faso [18] and acted as an information sharing forum for men in Nepal and South Africa [12],[19]. Furthermore, women in Uganda shunned PMTCT services because they were afraid of their partner¿s response to HIV positive test results and would rather seek for his approval prior to an HIV test [20],[21] despite antenatal HIV screening being fundamental to PMTCT of HIV [22].

To date, there is limited literature on the understanding of the relevance of MI in PMTCT services in the Malawian context. The objective of this study was to fill the gap in literature on MI by exploring the perceptions of men, pregnant women and health care providers on the importance and roles of MI in PMTCT services in Blantyre Malawi. Specifically, we explored the benefits of male involvement in PMTCT as well as the consequences of lack of male involvement in the services. Understanding the relevance of MI in PMTCT services from the users and service providers is critical to the development of programs that will enhance MI in PMTCT. This may contribute to increased adherence and retention in the Option B?+?regimen and the elimination of MTCT of HIV in Malawi.

## Methods

An exploratory descriptive qualitative study was conducted as part of a formative study, to gain understanding of the relevance of MI in PMTCT of HIV services in Blantyre, Malawi. The study was conducted among PMTCT service providers and users from December 2012 to January 2013. The study was conducted at the South Lunzu Health Centre (SLHC) and its catchment area. The SLHC was selected because it is a semi urban area which offered a less mobile community in the intervention trial informed by this formative study. The semi urban nature of the area also enabled the gathering of views that may be applied in both urban and rural settings [23]. This was part of the formative phase of an intervention trial which was later implemented in the same area. We conducted 6 key informant interviews (KIIs) with health care workers and 4 focus group discussions (FGDs) with men and pregnant women. Focus group participants¿ HIV status was not a criterion and we did not explore the participants HIV status. The FGD participants were not couples. Focus Group Discussions enhanced understanding of the relevance of MI among men and women of varying age groups [24] within the social context within which MI in PMTCT occurs [25]. Key Informant Interviews offered detailed information on the relevance of MI because they drew from the expertise of the informants [26]. Participants that refused participation in the FGDs cited time constraints as the main reason. We followed the RATS guidelines in reporting the results of this study (See Additional file 1: RATS Checklist).

### Selection and recruitment of male focus group participants

A total of 18 men, were conveniently sampled and recruited for the FGDs with assistance from health care workers. They were identified at the health centre and within the catchment area and were invited to attend the FGDs on a specific date and preferred venue. The men in FGDs were conveniently selected with variation in some variables such as age, place where they were identified a clinic or community, number of children and employment status. The men were further divided into two focus groups based on age and were classified as younger men within age range of 18-24 years and an older men age range of 25 and above. There were 8 participants in the younger men age group with 3 men who were non users and 5 were users of the health centre. Of the 5 users, only 2 had used the antenatal clinic. The older men age group had 10 participants with 5 men who were non users and 5 were users of the health centre. Of the 5 users 3 had used the antenatal clinic. Men were included in the study if they were able and willing to participate in FGDs, provided consent, were 18 years and above, and had wives with the youngest child below 5 years of age or had a wife who was pregnant at the time of the study. Our assumption was that this group of men would have some knowledge of PMTCT services and would actively contribute to the discussions. All discussants were literate and provided informed consent prior to the discussion.

### Selection and recruitment of female focus group participants

A total of 17 pregnant women attending antenatal clinic at SLHC were conveniently sampled to attend focus group discussions at the health centre. The research assistant and the Principal Investigator (PI) approached the women in the waiting area of the antenatal clinic to solicit interest in the study. Once a woman had shown interest, they were asked to remain after their antenatal clinic for informed consent procedures and the discussion. The female FGDs were further divided into two groups: younger age group with an age range of 18-24 years and an older group with an age range of 25+ years. Among the female participants, the younger female group had 9 participants while the older women age group had 8 participants. Women were included in the study if they were willing to participate in FGDs, ability to consent and had a male partner at home. The women also varied in parity. Variation allowed for exploration of themes that were common and different among varying groups [27]. All FGDs were conducted in a private room within the health centre. All discussants were literate and provided written informed consent prior to the discussion.

### Selection and recruitment of key informants

Health workers were purposively selected based on their role and responsibilities in PMTCT services at the clinic. We purposely included informants that were implementing the different aspects of PMTCT services at the clinic such as HIV testing and counselling, administration of ARVs and follow up on care, in order to broaden the responses across the scope of PMTCT services [27]. All the identified health workers accepted to participate in the study. The interviewees included one medical assistant, two nurse midwife technicians, two HIV Testing and Counselling counsellors and the PMTCT coordinator for Blantyre district. Interviews with health care workers were individually scheduled and were conducted within the health centre in a private room except for the PMTCT Coordinator¿s interview, which was conducted at her office at Blantyre District Health Offices. Inclusion in the study was preceded by written informed consent for participation from key informants. All Key Informants were literate and conversant in English and Chichewa languages.

### Interviews and discussion

Interviews with key informants and FGDs were guided by the following broad questions (Additional file 2: KII Guide and Additional file 3: FGD Guide):

a) What is the relevance of MI in PMTCT of HIV services?

b) What are the consequences of lack of MI in PMTCT of HIV services?

After the opening question; discussion ensued with an aim of getting more depth on the relevance of MI in PMTCT of HIV services. A pre-tested interview guide was used with the health care workers while a pre-tested discussion guide was used with men and pregnant women in FGDs. Interviews lasted for 45-75 minutes while discussions lasted for 60-90 minutes. All interviews and discussions were audio recorded using a digital voice recorder. The PI conducted all key informant interviews in English and Chichewa and facilitated the FGDs in Chichewa with assistance from 2 protocol-trained, one male and one female, research assistants. The study was stopped when there were no new ideas coming up which was characterised by participants offering the same responses. Audio recorded interviews were transcribed and translated into English by the PI as part of data familiarization and were verified by research assistants. The research assistants verified and proofread the transcribed and translated data against the recording for completeness of the transcripts and proofread the transcripts against the recorded interviews The PI then discussed points of divergence with the research assistants for the final transcripts.

Validity in the study was ensured by capturing verbatim accounts from the participants in their language, literal recording and detailed descriptions of the participants and use of a digital recorder to capture all the deliberations. We also employed constant comparison of the initial categories against all transcripts from the FGDs and KII. Key interpretations were checked by participants to ascertain validity at the end of each discussion or interview. The transcripts and coding were shared with other researchers to check if the codes were a better representation of the data. The credibility of the study was achieved through persistent inquiry during interviews to ensure that the issues under the relevance of MI were exhaustively discussed. The use of multiple methods like FGDs and KIIs in collecting data ensured reliability.

### Data analysis

Transcripts were exported to NVivo 9.0 for management and were analysed using a framework analysis approach. Themes were developed both from the research questions and the narratives as expressed by study participants [28]. Framework analysis was selected because it supports data description and explanations of a phenomenon [29]. Gale et al. [29] further states that framework analysis is under thematic analysis approach and aims at identifying common elements as expressed by participants before defining the relationship erupting from the data. We defined our cases as groups we interviewed such as women, men and health care workers. The steps in framework analysis as proposed by Gale et al. [29] and Pope et al. [30] are discussed in the context of this study.

#### Transcription

The recorded transcripts were transcribed and translated verbatim into English. The PI transcribed the data to be immersed in the study.

#### Familiarisation

The PI familiarised herself with the data by reading and rereading the transcripts, the notes and listening to the digital audio recorded proceedings to gain a full picture of the data before analysing it and enabled listing of key ideas.

#### Coding

One transcript was coded first. Codes, which were later grouped under themes, were identified as the transcripts were read through analysis of each phrase or line. We employed inductive and deductive coding. Coding systematically organised the data.

#### Developing an analytic framework

A set of codes that was developed from the previous stage were reviewed by an independent researcher who also coded the data. At this stage we decided on the codes to be used for the other transcripts.

#### Applying the framework or indexing

The codes identified were used for coding the rest of the transcripts whilst paying attention to any new codes that came out from other transcripts

#### Charting data into matrix

The identified themes were presented in a chart in Microsoft word and were applied to all transcripts in order to index the data. The chart was also used to compare the variance of ideas among the groups in order to identify areas of agreement and disagreement. The themes were verified to ensure that they were not over or under represented. In vivo codes that corresponded with the identified themes were highlighted. Themes were adjusted in order to realise more accurate themes and to avoid repeating data under several themes. Afterwards, codes were charted under the most appropriate themes thus reducing data through comparing and contrasting data and arranging similar data under the same theme.

#### Interpreting the data

All emerging interpretations were recorded in a notebook. At this point we determined the relationships among the themes and across the groups.

### Ethical approval

Prior to data collection, ethical approval was obtained from the University of Malawi¿s College of Medicine Research and Ethics Committee (COMREC-P 09/12/1279). The Blantyre District Health Office gave a written institutional support for the study. All study participants provided a written consent. Participants were assured of confidentiality to the extent possible with FGDs and interviews. All the interviews and discussions were conducted in a private room. Two research assistants, one female and the other male, were trained on the data collection procedures and both had prior human subjects¿ ethics training. The female research assistant is a research nurse with training in qualitative research methods and the male research assistant is a research community health educator trained in qualitative methods. Both research assistants and the PI were not members of staff at SLHC.

## Results

### Characteristics of focus group study participants

Socio demographic characteristics of the FGDs were reported earlier [23] however, in brief (Table 1): the age of the 17 female FGDs participants ranged from 18 to 41 years with a median age of 18 years. The gravidity ranged from 1-8 pregnancies with a median of 2 pregnancies. The age of the 18 male participants ranged from 20-33 years with a median age of 26.5 years. The age of the youngest child of the male participants ranged from 1-6 years with a median age of 2 years (Table 1).

**Table 1:** **Demographic characteristics of the study participants in the FGDs** (**N** =**35**)^**1**^

**Variable**	**Male**	**Female**
Sex	18	17
Median age (in years)	26.5 (20-33)	23 (18-41)
**Religion**		
?Roman Catholic	4	7
?Muslim	1	2
?Seventh day adventist	3	0
?Pentecostal	5	4
?Other	5	4
**Tribe**		
?Yao	2	5
?Ngoni	3	4
?Lomwe	8	6
?Other	5	2
**Education level**		
?Primary	5	9
?Secondary	12	7
?College	1	1
**Employment status**		
?Not employed	0	14
?Formal	3	1
?Self employed	15	2
**Age of youngest child(Males only)**		
?Pregnant wife	4	
?Under 1	6	
?1-?<?2 years old	1	
?2-?<?3 years old	2	
?3-?<?4 years old	3	
?4-?<?5 years old	2	

^1^Table one adapted from Nyondo et al 2014 [23]. Nyondo AL, Chimwaza AF, Muula AS: **Assessment of strategies for male involvement in the prevention of mother**-**to**-**child transmission of HIV services in Blantyre**, **Malawi**. *Global Health Action* 2013, **6**:22780.

### Characteristics of the Key informants

Sociodemographic characteristics of the key informants were: The age range was 24 to 42 years with a median age of 30.5 years. Five of the key informants were females with one male; four had college level education as highest level of education while the remaining two attained a secondary school as their highest education level.

### Relevance of male involvement in the prevention of mother to child transmission of HIV services

The major themes that emerged on the relevance of MI in PMTCT were a) uptake of interventions along the PMTCT cascade b) support mechanism and c) education strategy. Additional file 4 shows the distribution of themes according to participants¿ groups.

### a) Uptake of interventions along the PMTCT cascade

Participants in both FGDs and in KIIs stated that MI enhances uptake and adherence to PMTCT interventions, therefore minimising losses at the different cascades. Study participants stated that male involvement may influence the following sections: i) couple HIV counselling, ii) HIV testing, iii) uptake of ART and iv) management and follow up of pre ART participant.

#### Couple HIV counselling

Participants in both FGDs and KII stated that it is beneficial when partners are counselled together because they are presented with the necessary information for decision making on HIV testing at the same time. A male participant narrated how the counselling session is tailored for the couple as follows:

*They* (*health care workers*) *explain to you* (*a couple*) *on how it goes* (*procedures at the antenatal clinic for HIV testing*); *they ask if both of you have been* (*HIV*) *tested*, *if the response is no*, *they refer you for testing*; *if it is yes they counsel you appropriately. Everything that happens is tailored to the two of you*, *whatever is discussed relates to the two of you*, *and relates to how you can take care of yourself at home*¿¿*Older Male* (*OM*) *FGD*

Additionally, participants emphasised the benefits of couple counselling on HIV and were convinced that couple counselling would promote compliance to advice given because it eliminates mistrust that may arise when only one partner has been counselled. A male participant¿s response was:

¿.. *if they* (*husband and wife or partners*) *both come and all have received the necessary advice*, *when they get home*, *they will be practicing or following the advice without one questioning or doubting the other*¿..*because they were counselled at the same time*. (*OM FGD*)

Furthermore, it was stated that a couple benefits more from the expertise available through various professionals at the health facility. This was exemplified in the quote below:

*The advantage is that when you report to the health centre*, *there are different services*, *different professionals*, *such that the information one gets from the health workers is different from the information that a wife would share with a husband at home on the same topic*, *and one*¿*s questions will be answered which will enhance one*¿*s understanding*. (*OM FGD*)

#### HIV Testing

Participants in the FGDs reported that MI enhances uptake of HIV testing by the woman, her partner and their child. Key informants did not express this as an important factor for MI in PMTCT. Participants stated that an HIV test would be readily accepted when a couple presents themselves for PMTCT as opposed to times when a woman presents herself or with just her baby. The quote below illustrates this:

¿.*In most cases when both the wife and husband report to the antenatal clinic and are asked to take an HIV test*, *they will both accept to be tested*¿ (*OM FGD*)

Furthermore, female focus group participants and key informants reported that MI presents a man with an opportunity to be tested for HIV and that it would be easier for a man to take a test during that session as opposed to having his wife ask him to take a test outside of the health facility setting.

¿. *if we attend together and I get tested*, *the man may be encouraged and have an opportunity of getting tested as well so that we both know our* (*HIV*) *statuses. Older Women* (*OW*) *FGD*

#### Uptake of ART

Key informants stated that MI is essential, especially with the current PMTCT Option B + regimen whereby an HIV infected pregnant woman is initiated on ART immediately after diagnosis, because a man is made aware of the medication the woman and baby are taking. Key informants believed that MI would reduce the tendency of women taking drugs covertly particularly when a woman has not disclosed to her partner about her HIV positive status and consequent need for treatment. Key informants stated that taking ARVs overtly would be possible because MI would have created an opportunity for a facilitated disclosure of a couple¿s HIV status. Female FGDs participants further reported that it would be easier for a woman and her infant to initiate and remain on ART when a husband or father (to the baby) is involved because she would not be afraid of her partner¿s negative reaction. Additionally, KIIs reported that they insist on MI particularly in cases where a woman is HIV infected as a means of ensuring adherence to ARVs by the woman and also when she is refusing initiation of ARVs for PMTCT.

*In instances where a woman is refusing to start medication* (*ARVs*) *we also involve her husband because he is the person she usually resides or stays with and may be able to encourage her to start taking medications* (*ARVs*)¿¿*If the husband says that she can start* (*ART*), *most of them start*¿. *KII Respondent No 1*

Participants in FGDs stated compliance to ARVs will be well supported with MI because partners would remind each other on the dosage times, if on ARVs

*It* (*MI*) *is important so that we are able to remind each other* (*on dosage times*) *for instance*, *if one of us* (*within a couple*) *is infected and if we are counselled as a couple we will be able to remind each other* (*OW FGD*)

¿¿*suppose both* (*husband and wife*) *are positive*, *then it means both of you will be receiving medicine* (*ARVs*) *and will be taking them accordingly*¿.. (*OM FGD*)

The participants in both FGDs and KII stated that information relating to the care of the baby should be shared to both partners. This can mainly be achieved when male partners participate in PMTCT of HIV services. It was stated that it would be easier for a woman to implement PMTCT interventions on the child if her partner is aware of the interventions from the onset. Again, it was agreed that a male partner would be able to remind the woman the dosage times for the infants medication if the infant is on medication, or provide for the infants nutritional needs, should a couple opt against breastfeeding.

¿.*she may make decisions regarding herself on her own*, *but with the child she wants to seek permission from the baby*¿*s father*. (*KII Respondent no 3*)

¿¿. *a woman may not make a decision on her own*, *if we involve the man*, *it will be easier for the child to start ARVs earlier* ¿¿¿¿¿ (*KII Respondent no 4*)

### Management and follow up

Key informants stated that irrespective of whether a man initiates ART immediately or later, MI in PMTCT of HIV services offers health care providers an opportunity of managing and following up patients who are still in the pre ART phase. This theme was expressed by informants only.

¿ *if they are both* (*wife and husband*) *tested and we* (*health care workers*) *know the stage they are at*, *it is good because we are able to assess them as you know these days we follow up on our patients even those that are not yet on ARVs*, *it* (*MI*) *is a good thing*¿¿.. *KII Respondent No 1*

### b) Support system

Participants expressed that the MI in PMTCT creates a support system which was further categorised into a) positive living with HIV infection, b) creation of a fast track mechanism for women at the antenatal clinic c) family planning d) facilitated mutual disclosure and e) cultural appropriateness.

### Living positively with HIV infection

Female FGD participants reported that MI in PMTCT of HIV services creates a support system for the woman. It was stated that in cases of either HIV sero-discordance or concordance, support and encouragement from a spouse is necessary and vital for compliance to ARVs and for living positively with HIV. It was further stated that HIV positive sero concordant couples will support each other with treatment adherence, keeping clinic appointment dates as well as measures for preventing MTCT of HIV. Other forms of support would be in instances where partners would remind each other on the advice and counselling they received from the health care workers. The following quotes illustrate this:

*It is possible that sometimes*, *one* (*a woman*) *can be HIV infected*, *and one* (*a woman*) *is anxious and is quiet and is thinking about that*, *if he* (*male partner*) *is fine*, *he reassures you not to worry so long as one knows what one has. Younger Women* (*YW*) *FGD*

*A woman is initiated on antiretroviral therapy right there* (*after HIV diagnosis*). *There is this issue of denial*, *but when you have somebody and he is encouraging you* (*wife*), *you feel good that* ¿*Ok*, *I have nothing to do but to take this because my partner is agreeing*, *he is giving me support that I should do this*¿. *KII 04 Respondent*

### Fast track mechanism in antenatal care

Male participants in FGDs regarded their involvement as beneficial for women because it offers women an opportunity of accessing antenatal care faster without queuing up. Men observed that the practice at SLHC is for any woman who presents with a partner to be assisted first, thereby reducing their waiting time at the antenatal clinic. They further stated that women who report with their partners are treated with respect unlike those that report alone. The following quote showed this:

*For instance*, *when my wife came on Monday*, *there were 2 men who had accompanied their partners*, *all the women that came without their partners were left unattended*, *those that came with their partners were attended to first so that they do not delay*, *so my wife asked that she would like for me to be escorting her* (*for antenatal care services*), *and I accepted* ¿.*I accompany her so that she gets assisted quickly and I later continue with my other duties. Younger Male* (*YM*) *FGD*

Conversely, participants noted that women who attended the antenatal clinic without their partners experienced long waiting time before receiving antenatal care because health care workers prioritised women who came with their partners when rendering antenatal care.

¿¿ *a woman who has reported for antenatal clinic on her own*, *she takes time*, *they are taught in a group and it takes time even to get a test* (*HIV*), *comes back to the benches and queues up on the bench while waiting for her turn. OM FGD*

### Family planning

Female participants believed that through involvement, men would appreciate women¿s experience with pregnancy, labour and delivery and may be more agreeable to family planning or be satisfied with the number of children they have. Female participants believed that men insisted on having more children and subsequently refused family planning methods because they are not available during delivery to appreciate the process of labour and delivery. The following quote illustrates this

*It is important for them to be available in the delivery suite because we encounter problems*;¿¿ *and when you suggest to him to wait for 4*-*5 or 6 years before having another child*, *he refuses and tells you that he wants a child*, *because they do not know what happens in the labour ward. It is necessary for them to accompany us to understand what happens in there*, *it would cause them to be empathetic and agree on having a tubal ligation because the number of children is adequate. OW FGD*

### Facilitated mutual disclosure

Key informants stated that MI would promote facilitated and non-violent mutual disclosure of HIV status between partners. Key informants noted that some women have problems disclosing their HIV positive status results to their partners because they are afraid of negative reaction from them.

*When you have explained to them* (*a couple*) *very well*, *there are less problems*, *especially for women*; *sometimes when a woman comes and she is* (*HIV*) *counselled and* (*HIV*) *tested alone*, *and she is found* (*HIV*) *positive*, *when she goes home*, *others have come back and complained of marriage breakdown* ¿.. *so if these men come and receive the information together on what is needed*, *take part and agree*, *I think it will be good. KII 05 Respondent*

Female participants echoed that facilitated disclosure may avert possible marital tension secondary to an HIV positive result. The quotes below exemplify this:

*However*, *when it is only the woman who has come for an HIV test*, *she will only know her status without knowledge of the husband*¿*s status*, *it becomes difficult*, *in the event that the woman is HIV positive*, *and she has been asked to come with her husband*, *her husband may refuse and not come which creates room for arguments in their house because there is no unity. OM FGD*

¿..*when I* (*a pregnant woman*) *get home and explain to him* (*husband*), *he thinks that I am lying* ¿¿¿. *YW FGD*

### Cultural appropriateness

Participants in all FGDs and KIIs emphasized that MI in PMTCT of HIV services is important, particularly in the context of Malawian culture where it is established that a man is the head of the family. In view of that tradition, MI is fundamental because key decisions within a household are usually made by a man and in the same regard a woman may not make decisions about HIV testing and treatment in the absence of her partner or his consent. This is shown in the following quotes:

*It is very important because*, *in a family*, *the head of the family is the man and when the man is the head*, *he ought to take part in that. YM FGD*

*Yes*, *participation of men is important more especially in our culture men are the decision makers so if we leave them behind*, *it will not work but they have to be part of it. KII 04 Respondent*

*Because he is the one who is the head of the family so*, *if he takes part*, *he will be responsible for all the family affairs and the needs of his wife to prevent the child on PMTCT. KII 02 Respondent*

### c) Education strategy

There were two categories that came out under this theme and they are: health information and preventive measures.

### Health information

Participants in male FGDs reported that MI in PMTCT of HIV services is beneficial to the man because it presents him with information that will be helpful for his health and that of his family. Women participants in FGDs stated that they preferred to have men receiving health information directly from the health workers than women, especially if the information may negatively impact on their relationship. This information included the need for a woman to start ARVs immediately and need for infant prophylaxis.

*It* (*MI*) *is good because the man knows the significance and the reason for antenatal care and he is also aware of the doctors*¿ *advice because he is always there as well. OW FGD*

*It is important for a man to take part because when he goes to the hospital*, *he is informed of what may benefit him*, *the mother and child*, *if he follows that*, *the child may be born uninfected. YW FGD*

Key informants and participants in male FGDs stated that the information received forms the basis for the couples¿ decision regarding the number of children or how to ensure that the children are born HIV free despite HIV infection in both or one partner. The following quote illustrates this

*When you report to the clinic with your wife*, *it is a very helpful thing in your relationship because you can plan for your future together. OM FGD*

### Preventive measure

Another form of support expressed by men and key informants was the adoption of HIV prevention strategies. Key informants stated that men who are HIV infected may adopt preventive measures such as condom use in order to prevent transmission of HIV to their partners and their unborn child; men may decide to provide (milk) formula for their child in order to prevent MTCT of HIV. Key informants also stated that MI may potentially eliminate any behavioural problems present such as extramarital affairs. A couple would be aware of their problems and would take necessary measures to control or resolve the problem. The following excerpt illustrates this:

¿¿*however if both of you are aware of your statuses it means that the couple will be counselled together and will protect each other in the family*¿¿*OM FGD*

*It is important for a man to take part because when he goes to the hospital*, *he is informed of what may benefit him*, *the mother and child*, *if he follows that*, *the child may be born uninfected. YW FGD*

*Because we have enlightened the man on what HIV is*, *the dangers and all angles*, *including issues around bearing children*, *it enables the man not to contract HIV which may be passed on to the woman and the baby eventually. KII 04 Respondent*

### Consequences of lack of MI in PMTCT

This theme had two categories which were non-disclosure of HIV test results and non-compliance with PMTCT of HIV interventions (Additional file 4 shows the distribution of themes according to participants¿ groups)

### Non- disclosure of HIV status

Study participants in male FGDs and KIIs reported that the lack of MI in PMTCT perpetuates non-disclosure of HIV test results between partners consequentially affecting compliance with ARV regimen or any PMTCT interventions; in other cases a woman may not initiate ARVs for her or her baby. The following excerpts illustrate this

¿..*a woman sometimes keeps it* (*HIV positive status*) *as a secret and does not tell her husband that she is HIV positive*, *which causes them to take the drug privately. KII Respondent 02*

¿..*if she* (*a mother*) *has to give the child some ARVs then she will have to give it without the knowledge of the father*¿¿.. *KII Respondent 01*

### Noncompliance to PMTCT interventions

Participants in FGDs and key informants reiterated that the lack of MI may lead to non-compliance to ARV regimen. It was reported that sometimes women who may not have disclosed their HIV status to their partners find it challenging to comply with their ARVs and appointment dates.

¿*it becomes a problem because men are the heads of families and women abide by what the men says*; *as such it becomes difficult because they are afraid of the men*, *resulting in women doing something contrary from what they were told at the clinic. KII 06 Respondent*

## Discussion

The main finding of the study was that MI was reported to enhance the uptake and compliance with PMTCT interventions along the cascade. These findings are in line with other studies that reported that male involvement in maternal health services was perceived as a means for couple HIV counselling and testing [20],[31], increase uptake of ARVs for PMTCT and practice safe feeding measures [9],[32] uptake of an HIV test in women [20],[33] and other PMTCT interventions [34],[35] and reduced risk of mother to child HIV transmission [15].

Our study reported that MI in PMTCT creates an opportunity for individual men and couple HIV testing. This finding is in agreement with studies in Uganda, Kenya, and the Democratic Republic of Congo, where men felt that it promotes HIV Testing and Counselling (HTC) [36]¿[38] although some men within the Kenyan study viewed HIV testing as a deterrent for MI [37]. Human immunodeficiency testing is low in men even after accounting for HIV testing in women following PMTCT testing [39]. Participants believed that men have limited opportunities for HIV testing unlike women who apart from HTC services have PMTCT services as another avenue, hence offering early access to an HIV test for women [40]. Human immunodeficiency testing coverage in Malawi is higher for women than men [41] and more women in southern Africa access ARVs than men [42] thus highlighting the need for more avenues for HIV testing and treatment for men.

As reported by other studies, our study found that MI in PMTCT is an effective support system for pregnant women regardless of HIV status. Our findings agree with Kululanga et al, in Mwanza Malawi, who reported that MI in maternal health services was regarded as a ¿fast track¿ mechanism for women to attend ANC services [31]. In our study this was only reported by men, suggesting that women did not perceive it as such and also making us consider the understanding of men on the rationale for their involvement. The ¿fast track¿ mechanism is where women who attend ANC with male partners are attended to first. Although a ¿fast track¿ mechanism is beneficial, Kululanga et al reported that the ¿fast track¿ mechanism marginalises women without partners because it indirectly classifies them as women in a non-loving relationship or those that were impregnated outside of wedlock [31]. This strategy may contribute to client losses within the PMTCT cascade because these women may get lost without a health care worker noticing.

As in our study, the form of support of an HIV infected pregnant woman were reminders of PMTCT appointments, comforting the woman following an HIV positive result [43],[44] reminders to take ARVs, accompanying them to the PMTCT clinic and mutual support [45] and motivation to adhere to the preferred infant feeding option [44],[46]. Our study underscores what was reported by Mataya et al. [44] in their study in Malawi, where they stated that health care workers reported that women were non-adherent to their ARVs for PMTCT in the absence of male involvement while in cases where there was male spousal support, women were adherent. The support expressed in our study differed with that expressed by Iroezi et al. [45] in central Malawi, where the women highlighted that men were also supportive when women were scolded by other people within the community for taking ARVs. Furthermore, contrary to our findings on male support on choice of infant feeding, Bedell et al. 2014 [47] in Zomba, Malawi reported that women believed that they could independently decide on the feeding choice for the child, however MI was deemed relevant to other health care decisions for the baby.

Our finding of MI being culturally appropriate should be taken with caution because if it becomes a barrier for women to access HIV interventions as reported in other settings [20] then it contradicts the current Malawi HIV testing policy. The HIV testing policy in Malawi considers human rights by advocating for free access for all and emphasizes the provision of and ensuring access for women and girls to HIV interventions. The policy further alludes to protecting women from all forms of traditional beliefs that obstruct their health; however the practicality of that in the context of culture and gender may need further exploration [48]. Furthermore, Gruskin et al. [49] and King et al. [50] advocates for consideration of the health and human rights of pregnant women when providing HIV testing services by observing informed consent procedures, maintenance of privacy and confidentiality [51].

In agreement with other studies, this study reports that MI creates a health education channel for men on various issues. This finding supports what was reported in a South African study that men were interested in gaining more knowledge on PMTCT for them to better support their partners [52] as well as information on general antenatal care [37],[53]. Additionally, MI is a preventive measure against transmission of HIV secondary to the information shared. Similarly a study in Kenya reported that men stated prevention of transmission to the unborn child as a benefit for MI [15],[37]. As stated in our study, MI in PMTCT averts risky sexual behaviour and HIV transmission, which affirms the findings in a South African randomized study where MI reduced risky sexual behaviour [54].

Unique to our study, though, was that MI was reported to be a platform for a family to plan the future, such as limiting the number of children in the context of HIV infection. Contrary to this finding, a study in the same city of Blantyre reported that following an HIV uninfected child some HIV infected women desired more children [44]. The difference in the findings in the two studies may result from the scope of questions and objectives studied in both. However, this finding underscores the current proposals of integrating family planning services in PMTCT activities [55]¿[57] and may potentially prevent unintended pregnancies. Male involvement in PMTCT is in keeping with traditional values that confers authority to men in most households. This finding remains consistent with a study in Uganda, where society expects men to have authority over women and that HIV testing needed to be preceded by consent from a partner [38].

Male involvement facilitates amicable disclosure of an HIV status to one¿s partner because disclosure is facilitated by a trained counsellor. Although MI facilitates the mutual disclosure of HIV results [37],[58] through couple HIV counselling [33],[59], there is increased disclosure by men than women [34]. Disclosure is beneficial for positive living [60],[61] and adherence to ARVs [61]. While this study report of amicable disclosure following MI, other studies have reported of women experiencing violence following disclosure of HIV status results [62]¿[64] while other studies have stated that women refrained from disclosing their HIV status to avert violence [65], therefore, it is against this background that we emphasise disclosure strategies and prevention of intimate partner violence to be a part of MI in PMTCT.

On the other hand, we report that lack of MI facilitates non-disclosure of HIV positive test results. Other studies have also reported non-disclosure of HIV positive test results for fear of divorce and negative consequences [46],[58],[60],[63]. Non-disclosure may stem from the stigma associated with a positive HIV status [45]. Our study reported that some women do not disclose their HIV positive status to their partners for fear of abandonment. Similarly, marital tension arises as a consequence of HIV positive test results, particularly if the test was undertaken without the partner¿s knowledge [38]. We argue that with proper couple counselling and testing, facilitated disclosure, this problem may be minimised hence underscoring the relevance of MI in PMTCT of HIV programs. As in prior research, adherence to PMTCT of HIV interventions becomes a challenge in the absence of disclosure of one¿s HIV status to a partner [46].

## Conclusions

Male involvement is relevant for the uptake of interventions at the different cascades of PMTCT. The absence of male involvement may have negative effects on compliance with PMTCT interventions. Communities need education on the relevance of MI in PMTCT as well as alerting them on the consequences of lack of MI in PMTCT for the success of the service. Policy makers need to include both benefits of MI and the consequences of lack of MI in PMTCT of HIV services as part of the education strategy in order to strengthen MI.

### Study strengths and limitations

The strength of our study is that it presents the perceptions of service providers, pregnant women and men thereby offering a holistic approach to MI. This study is based on one health centre in Blantyre, Malawi and may not fully apply to other settings in Blantyre, Malawi. Nevertheless, the study highlights what are considered as the benefits and consequences of lack of MI in PMTCT which may be capitalised in programs aimed at enhancing MI. The results also highlight gaps that participants did not consider as relevant.

## Competing interests

The authors declare that they have no competing interests.

## Authors¿ contributions

ALN planned the study, developed study methods, interview guides and conducted the FGDs and KII, developed an analysis plan, analysed the data and drafted the manuscript. AFC and ASM supervised the planning, development of the methods, analysis plan, and data analysis and contributed and supervised the manuscript writing. All authors read and approved the final manuscript.

## Additional files

## Supplementary Material

Additional file 1: RATS Checklist. Click here for file

Additional file 2: Interview Guide for Key Informant Interviews. Click here for file

Additional file 3: Focus Group Discussion Guide. Click here for file

Additional file 4: Distribution of Themes on MI in PMTCT among Men, Women and Health care workers. Click here for file
