# Supplementary material for: Exploring the relevance of male involvement in the prevention of mother to child transmission of HIV services in Blantyre, Malawi
Source: BMC Int Health Hum Rights. 2014 Oct 30;14:30. doi: 10.1186/s12914-014-0030-y (PMC4422229; doi:10.1186/s12914-014-0030-y)
Supplement: Additional file 1: — RATS Checklist. [file s12914-014-0030-y-S1.docx]

| **ASK THIS OF THE MANUSCRIPT** | **THIS SHOULD BE INCLUDED IN THE MANUSCRIPT** | **Reference in manuscript** |
| --- | --- | --- |
| **R Relevance of study question** |  |  |
| Is the research question interesting? | Research question explicitly stated | The research question is explicitly stated at the end of the introduction section (pg. 5). |
| Is the research question relevant to clinical practice, public health, or policy? | Research question justified and linked to the existing knowledge base (empirical research, theory, policy) | The introduction section justifies the research question and the research question has been placed in the existing knowledge base (pgs.4-6). |
| **A Appropriateness of qualitative method** |  |  |
| Is qualitative methodology the best approach for the study aims?   - *Interviews:* experience, perceptions, behaviour, practice, process - *Focus groups:* group dynamics, convenience, non-sensitive topics - *Ethnography:* culture, organizational behaviour, interaction - *Textual analysis:* documents, art, representations, conversations | Study design described and justified i.e., why was a particular method (e.g., interviews) chosen? | The study design and the rationale for using Focus Group Discussions and Key Informant Interviews is justified (pg. 6) |
| **T Transparency of procedures** |  |  |
| *Sampling* |  |  |
| Are the participants selected the most appropriate to provide access to the type of knowledge sought by the study?  Is the sampling strategy appropriate? | Criteria for selecting the study sample justified and explained   - *theoretical:* based on preconceived or emergent theory - *purposive:* diversity of opinion - *volunteer:* feasibility, hard-to-reach groups | Key Informants were purposively selected while FGDs participants were conveniently sampled. The criteria and justification are on pages 6-8. |
| *Recruitment* |  |  |
| Was recruitment conducted using appropriate methods? | Details of how recruitment was conducted and by whom | The Principal Investigator (PI) and the research assistant recruited the pregnant women for focus group discussions at the antenatal clinic. The health care workers assisted in recruiting male participants for the male FGDs from the various departments at the health centre and in the catchment area. The clinic in charge assisted the PI in identification of Key informants at the health centre while the PI recruited the PMTCT coordinator from the Blantyre District Health Office. See page (6-8) |
| *Is the sampling strategy appropriate?* |  |  |
| Could there be selection bias? | Details of who chose not to participate and why | None of the identified Key informant refused participation. FGD participants that refused participation in the study cited time constraints as the reason for refusal. (see page 6 and 8). |
| *Data collection* |  |  |
| Was collection of data systematic and comprehensive? | Method(s) outlined and examples given (e.g., interview questions) | The methods followed in the study are outlined on pages 8-9. The interview guides are appended as additional files |
| Are characteristics of the study group and setting clear? | Study group and setting clearly described | The study group and setting are clearly described on pages 6-8 |
| Why and when was data collection stopped, and is this reasonable? | End of data collection justified and described | The period of data collection is stated on page 6. The reason for stopping data collection is on pg 9. |
| *Role of researchers* |  |  |
| Is the researcher(s) appropriate? How might they bias (good and bad) the conduct of the study and results? | Do the researchers occupy dual roles (clinician and researcher)? Are the ethics of this discussed? Do the researcher(s) critically examine their own influence on the formulation of the research question, data collection, and interpretation? | The researchers had no dual roles, see page 12. The ethical considerations in the study are described on page 11-12. The interpretation of the results is backed by participants quotes in the results section ( pgs. 13-24) |
| *Ethics* |  |  |
| Was informed consent sought and granted? | Informed consent process explicitly and clearly detailed | Informed consent procedures described under the Methods section on pages 7-8. |
| Were participants’ anonymity and confidentiality ensured? | Anonymity and confidentiality discussed | All quotes were anonymised. Data is stored securely in locked cabinets at the Malawi College of Medicine |
| Was approval from an appropriate ethics committee received? | Ethics approval cited | Ethical approval was granted and is cited on pages 11-12 in the methods section. |
| **S Soundness of interpretive approach** |  |  |
| *Analysis* |  |  |
| Is the type of analysis appropriate for the type of study?   - *thematic:* exploratory, descriptive, hypothesis generating - *framework:* e.g., policy - *constant comparison/grounded theory:* theory generating, analytical | Analytic approach described in depth and justified  *Indicators of quality:* Description of how themes were derived from the data (inductive or deductive)  Evidence of alternative explanations being sought  Analysis and presentation of negative or deviant cases | The data analysis approach is detailed on pages 10-11 under data analysis. |
| *Are the interpretations clearly presented and adequately supported by the evidence?* |  |  |
| Are quotes used and are these appropriate and effective? | Description of the basis on which quotes were chosen  Semi-quantification when appropriate  Illumination of context and/or meaning, richly detailed | Quotes presented under results (pg 14-24) and also as in the appended document that shows variation in responses among the participants. |
| Was trustworthiness/reliability of the data and interpretations checked? | Method of reliability check described and justified e.g., was an audit trail, triangulation, or member checking employed? Did an independent analyst review data and contest themes? How were disagreements resolved? | We employed constant comparison method during coding; codes were validated by an independent qualitative researcher. See page 10-11. |
| *Discussion and presentation* |  |  |
| Are findings sufficiently grounded in a theoretical or conceptual framework?  Is adequate account taken of previous knowledge and how the findings add? | Findings presented with reference to existing theoretical and empirical literature, and how they contribute | The findings are grounded in literature. Refer to pages 24-28 where we hav discussed our results in contrast and comparison with previous research in the area. |
| Are the limitations thoughtfully considered? | Strengths and limitations explicitly described and discussed | Described in discussion section (p.28-29) |
| Is the manuscript well written and accessible? | Evidence of following guidelines (format, word count)  Detail of methods or additional quotes contained in appendix  Written for a health sciences audience | The RATS checklist is included as appendix |
| Are red flags present? These are common features of ill-conceived or poorly executed qualitative studies, are a cause for concern, and must be viewed critically. They might be fatal flaws, or they may result from lack of detail or clarity. | *Grounded theory:* not a simple content analysis but a complex, sociological, theory generating approach  *Jargon:* descriptions that are trite, pat or jargon filled should be viewed sceptically  *Over interpretation:* interpretation must be grounded in "accounts" and semi-quantified if possible or appropriate  *Seems anecdotal, self evident:* may be a superficial analysis, not rooted in conceptual framework or linked to previous knowledge, and lacking depth  *Consent process thinly discussed:* may not have met ethics requirements  *Doctor-researcher:* consider the ethical implications for patients and the bias in data collection and interpretation | None |

*The RATS guidelines modified for BioMed Central are copyright Jocalyn Clark. They can be found in Clark JP:*How to peer review a qualitative manuscript*. In*Peer Review in Health Sciences*. Second edition. Edited by Godlee F, Jefferson T. London: BMJ Books; 2003:219-235*
