# Supplementary material for: Exploring the relevance of male involvement in the prevention of mother to child transmission of HIV services in Blantyre, Malawi
Source: BMC Int Health Hum Rights. 2014 Oct 30;14:30. doi: 10.1186/s12914-014-0030-y (PMC4422229; doi:10.1186/s12914-014-0030-y)
Supplement: Additional file 4: — Distribution of Themes on MI in PMTCT among Men, Women and Health care workers. [file s12914-014-0030-y-S4.docx]

**Additional File 4: Distribution of Themes on MI in PMTCT among Men, Women and Health care workers.**

| **Relevance of male involvement in PMTCT** | **Men** | **Women** | **Health Care Workers** |
| --- | --- | --- | --- |
| 1. **Uptake of Interventions along the PMTCT Cascade** |  |  |  |
| *Couple HIV counselling* | *** | *** | *** |
| *HIV testing* | *** | *** | *-* |
| *Antiretroviral Therapy Uptake* | *** | *** | *** |
| *Management and Follow up* | *-* | *-* | *** |
| 1. **Support System** |  |  |  |
| *Living Positively with HIV infection* | *-* | *** | *** |
| *Fast Track Mechanism* | *** | *-* | *-* |
| *Family Planning* | *-* | *** | *-* |
| *Facilitated Mutual Disclosure* | *-* | *** | *** |
| *Cultural Appropriateness* | *** | *** | *** |
| 1. **Education Strategy** |  |  |  |
| *Health Information* | *** | *** | *** |
| *Preventive Measure* | *** | *** | *** |
| **Consequences of Lack of male involvement in PMTCT** |  |  |  |
| 1. Non-Disclosure of HIV status | *** | *-* | *** |
| 1. Non-Compliance to PMTCT Interventions | *** | *** | *** |

**PMTCT= Prevention of Mother to Child Transmission**

*** = expressed it as a factor;**

**-= did not express it as a promoting factor**
